# Ecological and Epidemiological Consequences of Tick‐Control Interventions in Residential Neighborhoods: A Synthesis of The Tick Project

**DOI:** 10.1155/tbed/4940832

**Published:** 2026-01-22

**Authors:** Richard S. Ostfeld, Felicia Keesing

**Affiliations:** ^1^ Cary Institute of Ecosystem Studies, P.O. Box AB, Millbrook, 12545, New York, USA, caryinstitute.com; ^2^ Program in Biology, Bard College, Annandale-on-Hudson, 12504, New York, USA, bard.edu

## Abstract

Controlling populations of *Ixodes* ticks has emerged as a core strategy for reducing human exposure to tick‐borne infections. Several means of reducing the size of the tick population using chemical and biological acaricides show promise in field trials and are frequently used commercially in North America and Europe. The Tick Project (TTP) assessed whether the use of two commercially available methods of reducing the abundance of host‐seeking blacklegged ticks (*Ixodes scapularis*) reduced encounters with ticks and reported cases of tick‐borne disease in humans and their outdoor pets. Residential neighborhoods were the units of replication. Here, we synthesize the results of this large‐scale, long‐term ecological and epidemiological study and integrate them with comparable literature to assess: (1) the peridomestic risk factors linked to tick encounters and cases of tick‐borne disease; (2) the spatial scale of these risk and response factors; (3) discordance between ecological consequences of tick control (strongly reduced tick abundance) and weak or undetectable epidemiological responses; (4) possible causes of the failure of tick control to reduce disease incidence; and (5) future approaches to preventing tick‐borne disease with environmentally‐ and behaviorally‐based interventions. We conclude that the low efficacy of tick control in reducing cases of tick‐borne disease observed to date could be improved with greater attention to human behaviors that affect exposure risk.

## 1. Introduction

Human communities in temperate regions of North America, Europe, and Asia are experiencing increased incidence of diseases caused by pathogens transmitted by *Ixodes* ticks [1–5]. Foremost among these tick‐borne diseases is Lyme disease, caused by *Borrelia burgdorferi* and transmitted primarily by *I. scapularis*, *I. ricinus*, *and I. persulcatus* [6, 7]. These ticks also transmit the agents of other zoonotic diseases, such as babesiosis (*Babesia microti*) and anaplasmosis (*Anaplasma phagocytophilum*), adding to the public health burden.

Public health responses to epidemics of tick‐borne zoonoses have emphasized diagnosis and treatment, both of which remain problematic [8]. For example, serological tests for human infection with *B. burgdorferi* are subject to considerable rates of false‐negative and false‐positive results [9, 10]. And although treatment of infected patients with orally administered antibiotics within a few weeks of exposure is generally effective, antibiotic treatment failures are frequently reported [11].

Limitations of approaches to diagnosis and treatment have stimulated interest in the development of preventative measures. Prevention of tick‐borne infections could, in theory, be achieved by vaccination, but as of this writing, no vaccines against Lyme disease, human babesiosis, or human anaplasmosis are available for public use. Prevention is potentially enhanced by use of self‐protection methods such as tick repellents and protective clothing, but adoption of these methods can be inefficient, and incidence of tick‐borne diseases can be high even in populations for which these methods are readily available [12–17]. Recently, much attention has been devoted to controlling tick populations as a means of preventing transmission of tick‐borne pathogens to people and thus reducing disease incidence.

*Ixodes* ticks are vulnerable to several classes of insecticidal/acaricidal chemicals, including organophosphates, pyrethroids, and phenylpyrazoles [18]. Area‐wide spraying of these acaricides typically reduces the size of *Ixodes* populations by as much as 90%, depending on the timing, concentration, and method of delivery [19]. The potential for area‐wide spraying of these chemicals to reduce abundance of ticks provides the basis for a large and growing industry of pest‐control companies targeting ticks in residential areas where human and tick populations overlap [18]. Some companies offer alternative control products using “natural” acaricides for consumers concerned about toxicity or environmental damage from the typical chemical ingredients [6, 18, 20, 21]. Survey methods in regions of the United States reveal generally high levels of willingness by the public to adopt tick control as a means of reducing risk of tick‐borne disease [12, 14, 15, 17, 22, 23]. A recent study in a region of the United States with endemic Lyme disease showed high levels of public interest in environmental tick control and a high willingness by residents to pay an estimated USD50 to USD80 per year, depending on whether the control efforts were confined to their property or extended to the local community [24].

Willingness by occupants of Lyme disease‐endemic areas to adopt and pay for area‐wide deployment of acaricide and/or environmental management likely depends on the assumption that such control methods are effective in reducing risk of exposure to tick‐borne disease [24]. However, defining efficacy can be challenging. Efficacy can be measured by the ability of the intervention to reduce the size of the tick population, which is expected to correlate with the probability of a person encountering a tick. In turn, the probability of encountering a tick is expected to predict the probability of exposure to a given tick‐borne pathogen. However, only rarely are the consequences of efforts to control tick abundance pursued to estimate impacts on human‐tick encounters or cases of tick‐borne disease. Thus, the ability of people at risk of tick‐borne infections to use information, as opposed to untested assumptions, to evaluate their willingness to adopt and pay for acaricidal treatments is limited.

The Tick Project (TTP) was designed to evaluate whether tick‐control methods, deployed in residential neighborhoods within a Lyme disease‐endemic region, reduced human‐tick encounters and cases of tick‐borne disease for people and for their outdoor pets. TTP also included the conduct of systematic reviews of published literature and meta‐analyses to quantify the efficacy of recommended property‐management and household‐management actions purported to reduce risk of exposure to ticks and tick‐borne infections. Meta‐analysis was also used to assess the spatial scale (individual property, residential neighborhood, or larger areas used for recreation) at which risk‐reduction measures were most effective.

Here we provide a synthesis of the results of TTP’s fieldwork, lab work, and literature reviews to: (1) explore how features of the study design of TTP and related projects affect specific ecological and epidemiological outcomes, (2) evaluate the expected efficacy of direct control of ticks versus that of indirect control by managing habitat features, and (3) suggest designs of future efforts to prevent tick‐borne diseases using environmental approaches. A primary goal is to elucidate the efficacy of these methods and underlying causes of observed outcomes. The public’s willingness to pay for interventions intended to protect them from exposure to tick‐borne disease should be based on rigorous evidence for the efficacy of those interventions.

## 2. The Design of TTP

The fundamental question motivating TTP was whether tick‐control interventions targeted at *Ixodes scapularis* in residential neighborhoods protect human health by reducing human encounters with tick vectors and the incidence of tick‐borne disease. Tick encounters were defined as the detection of one or more ticks crawling on or attached to study participants. Residential areas were chosen, as opposed to areas used recreationally (e.g., parks, nature reserves), for two main reasons. One is that prior studies suggest that patients infected with tick‐borne pathogens encounter the tick(s) that transmitted the pathogens predominantly in residential areas [25, 26]. The second reason is that rigorous assessment of the epidemiological consequences of tick‐control interventions (disease incidence, tick encounters) over time requires sampling of human subjects known to experience habitats in which different experimental treatments and controls are imposed. Such sampling can be accomplished more effectively by enrolling subjects who reside in a specified residential area than those who sporadically visit recreational areas. Details of the experimental design are further described in [19, 27–29].

The spatial scale at which to deploy experimental treatments was informed by a seminal study conducted in three states of the northeastern United States [30]. This study used a randomized, placebo‐controlled, double‐masked design to ask whether a synthetic pyrethroid, bifenthrin, reduced the size of tick populations, human‐tick encounters, and incidence of tick‐borne diseases. Bifenthrin, or a placebo control (water), was sprayed on individual residential properties for one or 2 years, during which the properties were surveyed for ticks and the residents were monitored for diagnoses of tick‐borne illness. Despite a 63% reduction in abundance of ticks on bifenthrin‐treated properties compared to placebo control properties, no effects on human‐tick encounters or reported cases of tick‐borne diseases were detected [30].

One possible explanation for why reducing tick density within individual properties failed to affect tick encounters or cases of tick‐borne disease is that the residents may become exposed to infected ticks outside of their own properties, including elsewhere in their residential neighborhoods. TTP investigators conducted a meta‐analysis to quantitatively compare the evidence that exposure risk (tick encounters or reported cases of disease) was associated with environmental or behavioral factors measured at the level of the individual property, the neighborhood outside the individual property, or outside the residential neighborhood. Although significant odds ratios associating environmental or behavioral variables with exposure risk were detected at all three of these nested levels, the highest odds ratios were associated with the neighborhood scale [31]. These results supported the strategy of imposing tick‐control interventions at the scale of residential neighborhoods and using those neighborhoods, rather than the individual properties within them, as the units of replication [27].

The specific tick‐control interventions were chosen on the basis of likely efficacy in reducing tick abundance, safety, and availability for use by property owners. Both chemical and biological control agents can be effective in reducing the population size of blacklegged ticks, but both efficacy and safety vary [32, 33]. Chemical acaricides can have toxic effects on vertebrate animals and leave toxic residues in the environment [34, 35]. Given that most of these chemical products were developed as insecticides and repurposed as acaricides, the potential exists for non‐target effects on other arthropods. Whether acaricides are applied directly to hosts for ticks, as opposed to broadcasting them into the environment, is also expected to affect environmental and non‐target impacts.

TTP researchers selected two tick‐control methods that were commercially available. One was a biocontrol product (Met52) consisting of spores of the F52 strain of the fungus *Metarhizium brunneum*, deployed using a high‐pressure, truck‐mounted sprayer on the ground and low vegetation. In field trials, Met52 was found to have no significant effects on non‐target arthropods in either lawn or forest‐floor environments [34] and was thus considered a safe alternative to commercially available chemical sprays. The other method was TCS bait boxes, which consist of a small box containing a food bait to attract small mammals, which are important hosts for immature blacklegged ticks and the most efficient host species in infecting ticks with the agents of Lyme disease, babesiosis, and anaplasmosis [36–38]. Once they have entered the box through small holes, these small mammals are exposed to small quantities of fipronil, a chemical acaricide. Killing ticks on reservoir hosts is expected to reduce both the overall size of the tick population and also to selectively remove those ticks that would otherwise be most likely to become infected and therefore dangerous to people.

The study location was the highly endemic region of Dutchess County in southeastern New York State, where incidence of TBDs has been among the highest in the United States for many years [28]. Neighborhoods were defined as aggregations of roughly 100 adjacent residential properties separated by natural or built features (e.g., large parks or roadways) from other neighborhoods. Of a much larger set of candidate neighborhoods, TTP investigators selected 24 that had historically seen among the highest cumulative incidence of Lyme disease cases in the county in recent decades. Researchers systematically contacted as many residents of these neighborhoods as possible to enroll them in the multi‐year study, ultimately recruiting an average of 34% (range = 24%–44%) of properties and retaining > 85% throughout the study period. Agreeing to participate in the study meant providing the researchers with permission to sample ticks and small mammals on the property (seasonal efforts on a sample of properties) as well as answering survey questions about tick encounters and diagnosed cases of TBDs every 2 weeks.

**Figure 1:**
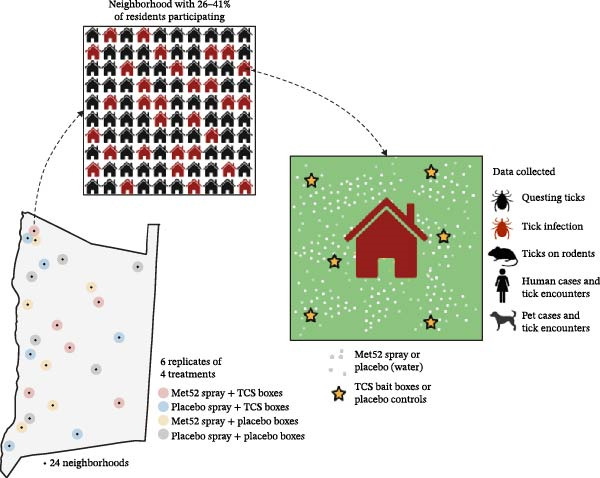
Schematic design of The Tick Project, in which 24 neighborhoods, all located in Dutchess County, New York, in areas of high incidence of tick‐borne disease, were randomly assigned to one of four treatment groups, as described in the main text. In each neighborhood of approximately 100 residential properties, 26–41% of residents agreed to participate in the study. Each participating home received the treatment assigned to that neighborhood, with Met52 spray (or a water placebo) sprayed on the vegetation and TCS bait boxes (or placebo boxes) placed around the property. Data were collected on the number of questing nymphal ticks, the prevalence of tick‐borne pathogens in those ticks, the number of larval ticks on rodents, and both human and outdoor‐pet encounters with ticks and cases of tick‐borne diseases. Treatment assignments were masked from participants and people collecting data. Locations of neighborhoods on the map are illustrative, as are locations of assigned treatments. Figure created in BioRender.

The treatment design included randomly assigning each of the 24 neighborhoods to one of four treatment categories, with six replicate neighborhoods in each category (Figure 1). The categories were active bait boxes (containing fipronil) and active Met52 spray; active bait boxes and placebo spray (pure water); inactive bait boxes (devoid of fipronil) and active Met52 spray; and placebo controls for both the bait boxes and spray. Met52 (or its placebo control) was sprayed twice each spring, a few weeks before and during the peak season of nymph activity. Bait boxes (or their placebo controls) were deployed in spring, replaced in early summer, and removed in mid‐autumn for the year. Treatments were imposed repeatedly each year for four consecutive years. The pest‐control company contracted to deploy the bait boxes and conduct the spraying was, of necessity, aware of which treatments were deployed in which neighborhoods, but none of the personnel collecting any of the data were aware of treatment categories in any neighborhood. Data on tick abundance and infection were collected by our research team during multiple visits to representative properties within each of the treatment categories. Data on encounters with ticks and diagnosed cases of tick‐borne disease were obtained from electronic or telephone surveys of all participating households every 2 weeks throughout the years of the study. None of the residents of the neighborhoods were aware of the treatment category for their neighborhood. Thus, the design was randomized, placebo‐controlled, and double‐masked [28].

## 3. Results of TTP

### 3.1. Control Neighborhoods

**Figure 2:**
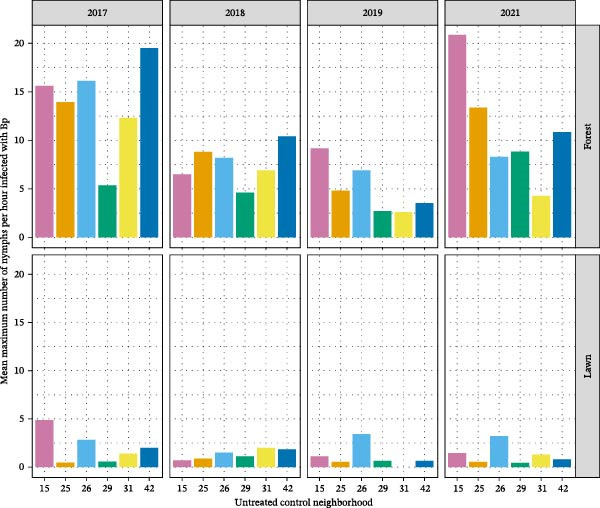
The density of nymphal ticks infected with *Borrelia burgdorferi*, the agent of Lyme disease, in six untreated control neighborhoods, averaged for two habitats, forest and lawn, across four years of data collection. The unmanipulated density of infected ticks (DIN) varied significantly across time, habitat type, and neighborhood. See [39] for additional details.

To provide a general context for the experimental treatments, we analyzed a suite of response variables (abundance of questing nymphs, infection prevalence of these nymphs with zoonotic pathogens, tick encounters, and reported cases of tick‐borne diseases) in the control neighborhoods alone. The nymph stage of this tick was selected because of strong evidence supporting bites by nymphs, as opposed to adult ticks, as the source of exposure to tick‐borne zoonotic pathogens [2, 6, 8]. This analysis provided a baseline level of variation between neighborhoods and between properties within neighborhoods to contextualize the impacts of treatments. We [39] found that some of the 24 neighborhoods consistently, over 4 years of sampling, had a higher density of nymphs (DON) than did others. Similarly, some of the properties within neighborhoods consistently supported a higher DON. The causes of these differences, however, were elusive; variation in DON between neighborhoods did not correlate with variation in the amount of forest cover or with the degree of forest fragmentation [39]. Nymphal infection prevalence (NIP) was measured only at the neighborhood scale because of insufficient samples from some individual properties. NIP did not vary across the control neighborhoods. In contrast, the density of infected nymphs (DIN), which is the product of DON and NIP, did vary significantly by neighborhood, habitat (forest vs. lawn), and over time (Figure 2). For these control neighborhoods, we did not observe any significant correlation between DON and human encounters with ticks nor between DON and cases of TBD in either pets or humans. We suggest that the causes of between‐neighborhood and between‐property variation in DON are worth further pursuit, as is the relevance of this spatial variability in tick abundance for human risk of exposure. The observed variation between properties and between neighborhoods in DON, which we suspect is typical of Lyme disease‐endemic areas, decreases the likelihood of detecting significant effects of interventions and thus poses a challenge to study designs. However, it also provides opportunities to enhance designs to account for spatially variable risk at small to moderate scales.

### 3.2. Consequences of Interventions for Tcks and Pathogen Prevalence

Across all the neighborhoods in all four treatment categories, DON was approximately one order of magnitude greater in forest than in lawn (Figure 3), and DON in shrub/garden habitat was intermediate [28]. The deployment of TCS bait boxes was associated with a ~50% reduction in DON in both forest and lawn habitats, as compared with placebo bait boxes [28] (Figure 3). The effects of TCS bait boxes were initially observed in the first year of their deployment, a result that was unexpected given that the bait boxes are designed to target larval ticks on small‐mammal hosts, with impacts on DON expected the subsequent year [29]. These impacts of TCS bait boxes in reducing DON remained consistent over time and did not increase in strength. Cumulative effects would be expected if the treated locations were isolated from nearby, untreated areas. The absence of detectable cumulative effects suggests that nearby, untreated areas might have acted as sources of immigration for ticks each year, such that the impacts of the bait boxes in each year were independent of prior years. Neighborhoods with active Met52 spray had slightly lower DON than did those with placebo spray, but this difference was not significant. We observed no additive or interactive effect of the two interventions when they were deployed together, reinforcing the conclusion that the effect of Met52 was weak to absent [28]. We observed no changes over the years of treatment in the impacts of Met52 [29].

Figure 3The mean density of nymphal ticks (DON) as a function of the four treatments in (A) forest and (B) lawn habitats across the four years of sampling. Treatments are indicated by their active component, so that Control indicates a full placebo treatment, and Bait boxes indicates active bait boxes but placebo Met52 spray. Error bars indicate± one standard error of the mean.

**Figure:**
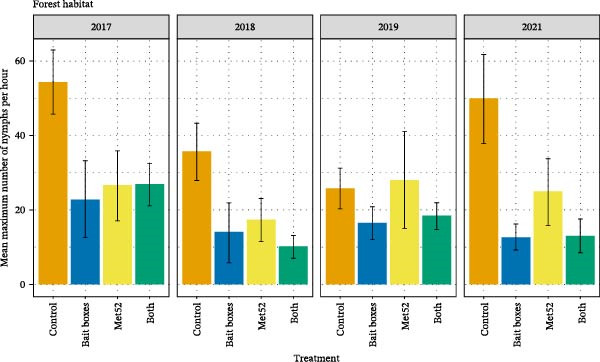
(A)

**Figure:**
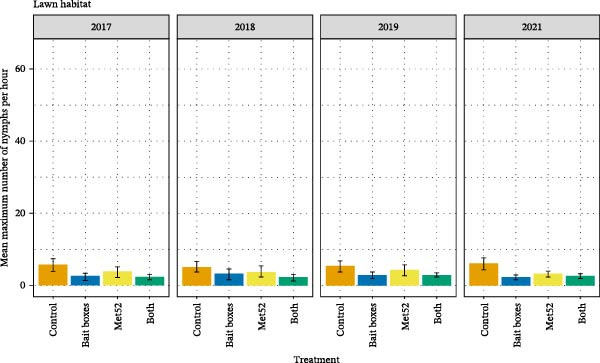
(B)

**Figure 4:**
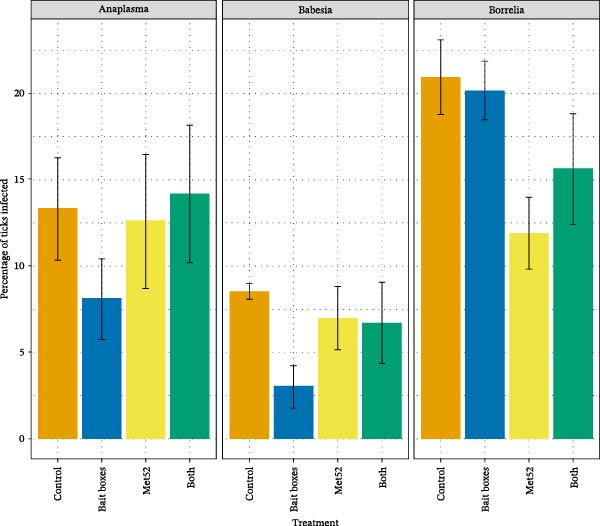
The mean percentage of nymphal ticks infected (NIP) with each of three tick‐borne pathogens, *Anaplasma phagocytophilum, Babesia microti*, and *Borrelia burgdorferi*, as a function of the four treatments applied to neighborhoods in The Tick Project. Treatments are indicated by their active component, so that Control indicates a full placebo treatment, and Bait boxes indicates active bait boxes but placebo Met52 spray. Error bars indicate ± one standard error of the mean. A detailed analysis of these results, along with probabilities that ticks were coinfected with multiple pathogens, is provided in [19, 40].

Contrary to expectations, NIP with the three most prevalent pathogens, *B. burgdorferi*, *B. microti*, and *A. phagocytophilum*, was not reduced in neighborhoods treated with TCS bait boxes, as compared to control neighborhoods ([19]; Figure 4). In neighborhoods treated with active Met52, NIP for *B. burgdorferi* was significantly lower than in the control neighborhoods; however, no differences between active and control neighborhoods were observed for NIP with the two other pathogens [19] (Figure 4). For NIP with *B. burgdorferi*, we observed a significant, cumulative reduction in NIP over the years of interventions, although such a reduction was not observed for the other two pathogens [19]. Variability between years in NIP for all pathogens unrelated to the treatments was observed, and pursuit of the potential causes of this variation seems worthy of further study.

Coinfection of individual ticks with two or more zoonotic pathogens is frequently observed and relevant to the diagnosis and treatment of tick‐borne diseases [41]. Overall, coinfection rates of nymphal ticks collected during TTP activities were similar to those reported in endemic areas of the northeastern United States [40]. Small rodents and shrews are the most competent reservoirs for *B. burgdorferi*, *B. microti*, and *A. phagocytophilum* [37, 41, 42]. As a consequence, acquisition of multiple zoonotic pathogens by ticks during their larval blood meal is significantly more likely to occur for larvae feeding on small rodents and shrews as compared to larvae feeding on other hosts [41]. Consequently, we expected that deployment of TCS bait boxes, which selectively kills larval ticks feeding on small rodents and shrews, should reduce prevalence of coinfection in nymphal ticks. Our results supported this expectation. In control neighborhoods and those treated with active Met52, rates of coinfection were higher than expected and similar to levels we observed in prior studies without tick‐control interventions [40]. In contrast, the bias toward coinfection was eliminated in neighborhoods treated with TCS bait boxes [40].

### 3.3. Consequences of Interventions for Human‐Tick Encounters, Pet‐Tick Encounters, and Cases of Tick‐Borne Diseases in Humans and Outdoor Pets

Despite the ~50% reduction in DON in neighborhoods receiving TCS bait box treatment, we observed no effect of this treatment, nor of Met52 or both together, on reported encounters of human or outdoor‐pet residents with ticks [28]. Reduced tick numbers and coinfection prevalence associated with the active TCS bait box treatments were similarly not associated with reduced reported incidence of tick‐borne disease in human residents [28] (Figure 5). However, a significantly reduced incidence of tick‐borne disease in outdoor pets was observed in neighborhoods treated with TCS bait boxes. No reductions in disease incidence in either humans or pets were associated with active Met52 treatments [28] (Figure 5).

Figure 5Cumulative number of cases of tick‐borne disease in (A) people and (B) outdoor pets in relation to neighborhood treatments in The Tick Project. Case numbers are based on participant reporting. Error bars represent± 1 standard error of the mean. Treatment did not significantly affect the number of self‐reported cases for people, but neighborhoods treated only with placebos had significantly more cases in outdoor pets than neighborhoods treated with any of the active methods. For details, see [28].

**Figure:**
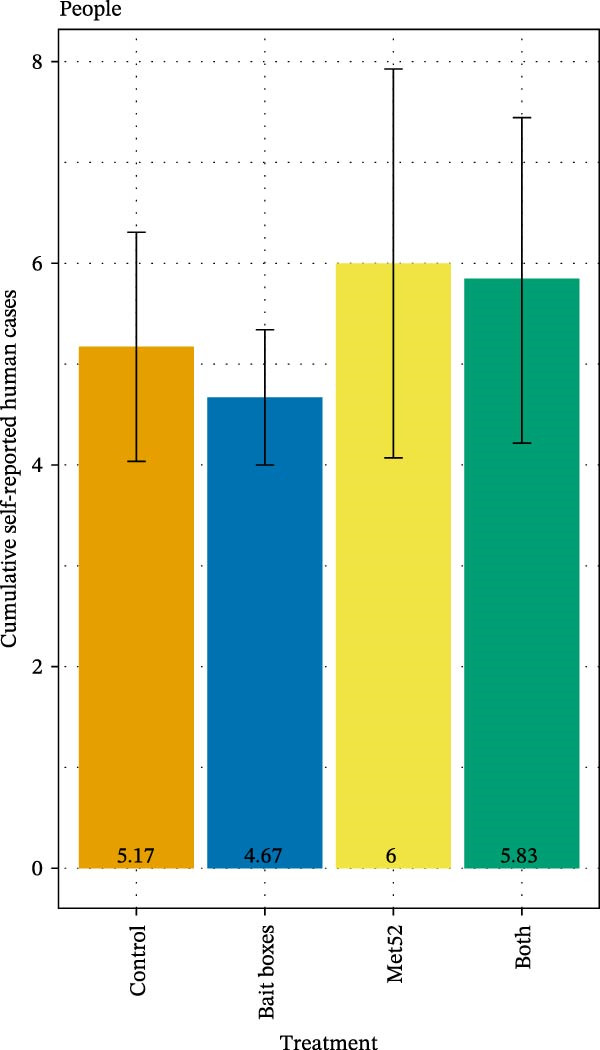
(A)

**Figure:**
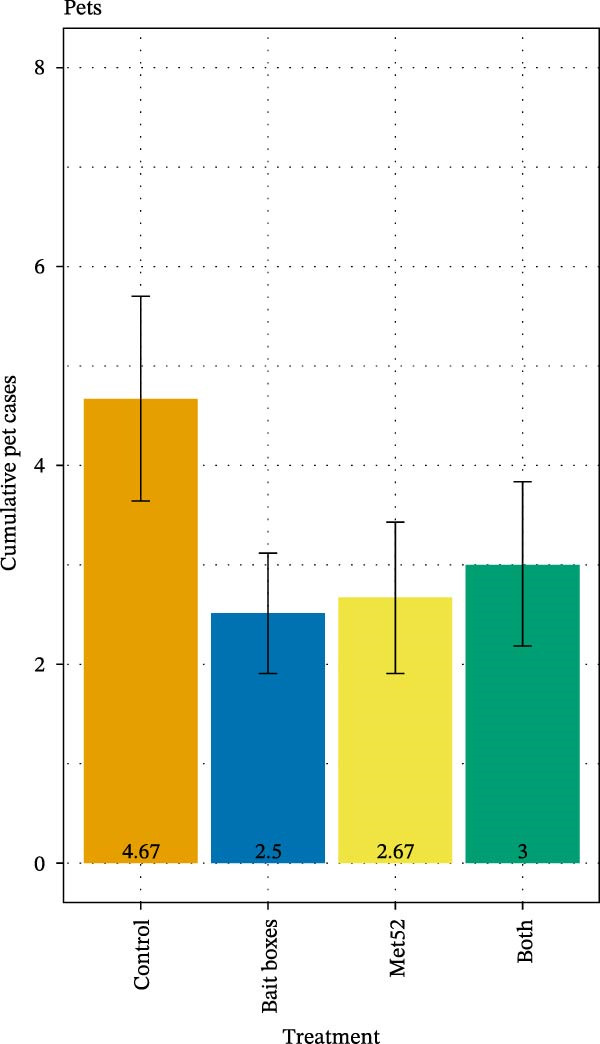
(B)

## 4. Discussion

TTP was undertaken to expand recent efforts to determine whether controlling ticks in residential areas produces direct health benefits in local populations. One of the two tick‐control interventions, TCS bait boxes, was associated with a strong, significant reduction in density (but not in infection prevalence) of nymphal blacklegged ticks (DON), which is the life stage responsible for the great majority of tick‐borne illnesses in eastern North America. This reduction in DON, however, was not associated with reduced reported incidence of tick‐borne disease in the human residents of the residential neighborhoods in which the interventions were imposed. The reduction in DON associated with this intervention similarly failed to decrease reported human encounters with ticks. In contrast, outdoor pets in neighborhoods treated with TCS bait boxes experienced significantly lower incidence of tick‐borne disease compared to control neighborhoods.

In the absence of vaccines, tick control is a hallmark of preventative measures intended to reduce the incidence of tick‐borne disease. Both chemical and biological acaricides, deployed in the habitats where ticks occur as well as targeted directly to vertebrate hosts, are capable of causing excess tick mortality and reducing tick population size [32, 43, 44]. Studies designed to assess the efficacy of acaricidal treatments on natural populations of ticks typically find strong reductions in tick abundance that reinforce the potential for such treatments to protect human health. It is possible, however, that some design features of these studies challenge their ability to assess likely epidemiological outcomes. As discussed in Ostfeld and Keesing [45], designs of field efficacy studies frequently do not randomize the selection of sites for imposing treatments versus controls. Randomization helps to reduce the potential impacts of prior conditions, whether or not those conditions are quantified, on the response to experimental treatment. Controls for the acaricidal treatment often consist of the absence of acaricide, rather than a placebo. Even when a placebo control is used, investigators collecting data on tick abundance are often not masked (“blinded”) to which treatment was imposed on the sites in which they are working. When human subjects are incorporated to assess health consequences of interventions, masking these subjects in addition to researchers (double‐masked, or double‐blind) is important for experimental rigor. Randomization, placebo controls, and masking are desirable for reducing or eliminating the potential for implicit bias to affect the results of the studies, thus strengthening the design. These design features can be logistically difficult and expensive, but the differences in efficacy between studies with and without them [45] suggest that they are important to include whenever possible.

We are aware of only three major initiatives that have pursued the consequences of field studies using acaricidal treatments for human‐tick encounters and incidence of tick‐borne disease [28, 30, 46]. Considerable reductions in DON were observed in two of these studies [28, 30] but not from the third [46]. However, none of the studies found a corresponding reduction in reported human‐tick encounters or human incidence of tick‐borne disease. Including these epidemiologically relevant response variables necessarily introduces human subjects into the study design, adding logistical and financial challenges. Nevertheless, data on these variables are required to assess human health impacts of tick control. Efforts to identify new chemical or biological agents capable of controlling ticks are important, but should be accompanied by or followed with studies of impacts on human‐tick encounters and human incidence of tick‐borne disease.

A notable result from TTP was that reported cases of tick‐borne disease in outdoor pets were significantly reduced in neighborhoods receiving the TCS bait box treatment [28]. The significant effect of bait boxes on outdoor animal, but not human, inhabitants of the treated neighborhoods was observed despite the somewhat small sample size of outdoor pets in our study [28]. We hypothesize that an effect of tick reduction on reported TBD cases in pets, but not in humans, could be caused by differences in space use by the two groups. Although ~50% reductions in DON were observed in both lawn and forest habitats, overall tick densities were ~10X higher in forests [28, 39]. If people spend most of their outdoor time in lawn and garden areas, the effects of a 50% reduction in DON in an already low‐risk area may not manifest as reduced human incidence. If outdoor pets instead spend more outdoor time in forested areas, the same relative decrease in DON may be more meaningful for disease reduction. Gathering more information on how people and their pets use different habitats in residential areas is an important research frontier.

If the relationship between tick abundance and the probability of human encounters with ticks, leading to potential infection, is strongly nonlinear, it is possible that moderate decreases in tick abundance might only marginally decrease exposure risk. We are unaware of a mechanism that might underlie such a threshold effect but suggest that further research could address the shapes of the association between tick abundance and human encounter probability. Our study took place in a county that has been experiencing substantial incidence of Lyme disease and other tick‐borne diseases for several decades and in which public awareness of ticks and tick‐borne diseases is relatively high [47]. If accompanied by frequent use of self‐protective measures, such as repellents, protective clothing, and avoidance, reduced tick abundance might be expected to have only modest health impacts beyond those of other mitigations of risk. (Note that enrollment in TTP was limited to households that did not apply area‐wide acaricides or insecticides independent of our research [28].) Reduced efficacy in areas with historically high disease incidence seems paradoxical, given that high‐incidence areas are generally a high priority for tick‐control interventions. We suggest that high‐incidence areas without a history of illness that stimulates other mitigations, i.e., those recently invaded by ticks, might experience the highest benefits of tick‐control interventions.

Direct acaricidal control of tick populations might be less powerful in reducing incidence of TBDs than are indirect measures, such as managing property features that affect hosts for ticks and/or abiotic features that influence tick survival. Property management actions such as the removal of brush or woodpiles, addition of desiccating barriers between forest and lawn, and installation of wildlife fencing are frequently recommended by state and federal health agencies. However, our meta‐analysis of the published effects of such actions on human‐tick encounters and cases of tick‐borne diseases failed to support their efficacy. Paradoxically, the risk of tick bites and TBD tended to increase (odds ratio significantly > 1) with increasing landscape‐related tick‐control measures, such as clearing brush, trimming branches, and placing a dry barrier between lawn and woods [48]. A recent study of 16 matched pairs of fenced vs. unfenced residential properties in CT, RI, and NY found no significant effects of wildlife fencing on encounter rates with adult or nymphal *I. scapularis* ticks, nor on infection prevalence with zoonotic pathogens [49]. Property management actions in general appear to have inconsistent and sometimes counterproductive impacts on the risk of tick encounters and disease incidence. Knowledge by the public of the efficacy of past studies of both direct and indirect tick‐control actions might alter the willingness to pay for future interventions.

Low effectiveness of direct and indirect tick control in reducing human encounters with ticks and exposure to tick‐borne pathogens might justify a renewed focus on increasing compliance with recommendations regarding personal protection. Unfortunately, a recent review of the efficacy of personal protective measures, such as use of repellents, protective clothing, and tick checks, indicated that none of the widely recommended measures consistently reduced risk or incidence of tick‐borne disease in the United States [16]. Small samples and inconsistent study designs present a challenge in drawing general conclusions from the individual studies [16], and therefore, it remains possible that some measures, singly or in combination, could be protective. The potential for interactive effects of tick control and personal protective measures in reducing exposure to tick‐borne diseases provides an opportunity for new approaches.

A vaccine based on Outer Surface Protein A of *B. burgdorferi* was briefly available in the early 2000s but was withdrawn from the market. This vaccine against Lyme disease, and others currently undergoing evaluation, will not protect patients against other tick‐borne pathogens, which constitute a growing health threat. Vaccines that confer resistance to tick bites, rather than to specific tick‐borne pathogens, are being pursued but have not, to our knowledge, reached the clinical trial stage. Vaccines found to be safe and effective still confront complex questions regarding public acceptance, the geography of distribution, and the potential for relaxed vigilance against ticks in vaccine recipients.

The pursuit of preventative measures against tick‐borne diseases has been accelerated by well‐known challenges in diagnosing and treating these illnesses. It seems ever clearer that both pre‐exposure and post‐exposure interventions need to be vigorously pursued and improved to reverse the growth in incidence of tick‐borne infections. Understanding the epidemiological impacts of interventions is critical and, as recent studies have shown, cannot be inferred from demonstrated impacts on tick abundance and infection.

## 5. Conclusions

TTP tested the hypothesis that reducing the abundance of tick vectors within residential neighborhoods would decrease reported cases of tick‐borne disease in the residents of those neighborhoods. The 50% reduction in abundance of questing nymphal ticks (*Ixodes scapularis*) did not reduce human‐tick encounters or cases of tick‐borne disease, although cases of disease in outdoor pets were reduced compared to placebo controls. Potential causes of the failure of tick control to protect residents from tick‐borne disease include strongly nonlinear relationships between tick numbers and human exposure risk, human avoidance of tick‐infested microhabitats in their yards and neighborhoods, and use of protective clothing and repellents that prevent tick attachment even where hazard is high. These data, combined with meta‐analyses of risk factors, suggest that some widely recommended behavioral interventions, e.g., removing brush and excluding deer, are not effective. Despite studies showing a public willingness to pay for tick control in Lyme disease‐endemic regions, TTP and comparable randomized, placebo‐controlled, double‐masked experiments aimed at tick control suggest that the expectation of health protection from tick control may not be achieved. Similarly, efforts to discover new chemical or biological control agents for ticks should assess efficacy in reducing incidence of tick‐borne disease. We conclude that human behavioral data should be incorporated into future assessments of pre‐exposure interventions and that these should be combined with improved post‐exposure treatments.

## Funding

The Tick Project was supported by the Steven and Alexandra Cohen Foundation, the US Centers for Disease Control and Prevention, New York State, the Dutchess County Water and Wastewater Authority, the Ian Mactaggart Trust, the John Drulle, MD Memorial Lyme Fund, Inc., the Pershing Square Foundation, the Walbridge Fund, Nina Brown deClercq, Susan and Jim Goodfellow, Elyse Harney, Eric Roberts, and Pamela and Scott Ulm.

## Conflicts of Interest

The authors declare no conflicts of interest.

## Data Availability

No new data were presented in this manuscript.
